# ECG Noise Cancellation Based on Grey Spectral Noise Estimation

**DOI:** 10.3390/s19040798

**Published:** 2019-02-15

**Authors:** Shing-Hong Liu, Cheng-Hsiung Hsieh, Wenxi Chen, Tan-Hsu Tan

**Affiliations:** 1 Department of Computer Science and Information Engineering, Chaoyang University of Technology, Taichung 41349, Taiwan; shliu@cyut.edu.tw; 2 Biomedical Information Technology Laboratory, The University of Aizu, Fukushima 965-8580, Japan; wenxi@u-aizu.ac.jp; 3 Department of Electrical Engineering, National Taipei University of Technology, Taipei 10608, Taiwan; thtan@ntut.edu.tw

**Keywords:** ECG noise cancellation, power line noise, electromyogram noise, empirical mode decomposition, ensemble empirical mode decomposition, grey model, grey spectral noise estimation

## Abstract

In recent years, wearable devices have been popularly applied in the health care field. The electrocardiogram (ECG) is the most used signal. However, the ECG is measured under a body-motion condition, which is easily coupled with some noise, like as power line noise (PLn) and electromyogram (EMG). This paper presents a grey spectral noise cancellation (GSNC) scheme for electrocardiogram (ECG) signals where two-stage discrimination is employed with the empirical mode decomposition (EMD), the ensemble empirical mode decomposition (EEMD) and the grey spectral noise estimation (GSNE). In the first stage of the proposed GSNC scheme, the input ECG signal is decomposed by the EMD to obtain a set of intrinsic mode functions (IMFs). Then, the noise energies of IMFs are estimated by the GSNE. When an IMF is considered as noisy one, it is forwarded to the second stage for further check. In the second stage, the suspicious IMFs are reconstructed and decomposed by the EEMD. Then the IMFs are discriminated with a threshold. If the IMF is considered as noisy, it is discarded in the reconstruction process of the ECG signal. The proposed GSNC scheme is justified by forty-three ECG signal datasets from the MIT-BIH cardiac arrhythmia database where the PLn and EMG noise are under consideration. The results indicate that the proposed GSNC scheme outperforms the traditional EMD and EEMD based noise cancellation schemes in the given datasets.

## 1. Introduction

The wearable device has been widely studied in recent years. In the related researches, it is essential to monitor the cardiac and physical activities in users with congestive heart issues at home. The electrocardiogram (ECG) is an important signal in monitoring the cardiac activity. A Holter ECG device is frequently applied to record long-term ECG signals and helps to find arrhythmic heartbeats within twenty-four hours [1]. Another apparatus is called an Event Recorder that is able to record one-minute ECG signals when a user feels uncomfortable on the chest region [2]. Currently, the commercial Holter ECG apparatus or Event Recorder only record the ECG signals, and is lacking in the real time analysis of the ECG signals. Moreover, the user has been encouraged to neither do any severe exercises nor take a shower, because these two kinds of devices do not have a high ingress protection rating.

An ECG patch is a wearable device, which not only records ECG signal, but also shows some cardiac information on the smart phone in real time [3]. However, its function for the arrhythmic beat detection is not better than the ECG Holter analysis. The problem is that the ECG patch is used under a body-motion condition, which is easily coupled with some noise, like motion artifact and electromyogram (EMG). Therefore, how to cancel the noise in real time would help to develop the applications of ECG patch. In the measurement of ECG signals, it is vulnerable to various noises and interferences, such as the EMG, 50 or 60Hz power line noise (PLn), baseline drift and measurement noise and so on. In [4,5], it was found that for complex QRS wave of a standard ECG signal, its power is within the frequencies lower than 30Hz, and its peak power is within the range between 4 Hz and 12 Hz. Generally, the high frequencies in the PLn and EMG noise are able to cover up the characteristic of an ECG signal such that the QRS wave group cannot be located accurately [5]. Besides, it is known that ECG signals affected by noises will lead to erroneous diagnosis by physicians. Therefore, it is crucial to remove the PLn and EMG noise in the ECG monitoring and clinical diagnosis.

Traditionally, ECG noise cancellation methods applied a low-pass filter [6] to remove the high-frequency components in noise while a high-pass filter and adaptive filter are used to rid of low-frequency vibrations, such as baseline drift [7,8] and respiratory interference [9]. Since most of the noises in ECG signals are distributed at the high-frequency components, and the traditional low-pass filters cannot solve the problem that signal and noise co-exist within the same bandwidth. Augustyniak [10] used the time-frequency transform to rescale the ECG coupled with different noises and then eliminated the noises to enhance the signal noise ratio. Rundo et al. [11] applied the bio-inspired nonlinear system to cancel the noise in an ECG signal and then to align the ECG and pulse wave. Some ECG denoising methods employ the frequency decomposition technique, such as the wavelet transform [12], the empirical mode decomposition (EMD) [13] and the ensemble empirical mode decomposition (EEMD) [14]. The EMD is a pre-processing algorithm of Hilbert-Huang Transform (HHT) which was introduced by Huang et al. [15] with a capability to perform time-frequency transformation for non-linear and non-stationary signals. In recent years, the EMD has been widely applied in biomedical signal processing, such as ECG [13,14], EMG [16] and blood pressure waves [17].

The basic idea for ECG noise cancellation consists of two main stages. First, an ECG signal is decomposed by the EMD into a set of IMFs through which the noisy IMFs are discriminated and discarded. Second, the ECG is reconstructed by the retained IMFs. By this doing, the noise in the ECG signal is reduced or cancelled. To be successful in the EMD-based noise cancellation, a fundamental problem is to estimate the noise energy appropriately in each IMF. In addition, the problem of mode mixing occurs during the EMD decomposition. The problem of mode mixing comes from either an IMF mingled with signals of different scales or other IMF appeared in the combination of other components. To overcome the mode mixing problem in the EMD, the EEMD [18] was proposed with an additive white noise into the ECG signal. The EEMD is able to reduce the mode mixing effect on the next IMF scale. The EEMD has been widely applied in noise cancellation. For example, Chang et al. presented a scheme to cancel the white noise in ECG signals where noisy low-order IMFs were removed by a predefined threshold [12]. Jenitta et al. used the zero-crossing ratio of adjacent IMFs to discriminate noisy IMFs by its noise energy [19]. Yannis et al. proposed a scheme according to the energy of the first-order IMF through which noise cancellation was performed among IMFs for ECG signals [20]. Kumaravel et al. presented a genetic algorithm to determine the noise energy threshold in the first-order IMF for the PLn cancellation in ECG signals [21].

The methods described above generally deal with white noise and the PLn only. Besides, All of them estimates the noise energy in the first-order IMF. However, only considering the noise energy in the first-order IMF might not be appropriate especially when the signal energy of the related IMF is high. This will lead to over-cancellation and result in distortion in the reconstructed ECG signal. Consequently, this paper considers noise energies in every IMFs through the noise magnitude spectrum.

As we know, very few approaches to ECG noise cancellation are based on noise magnitude spectrum. In this paper, two-stage discrimination scheme is proposed to estimate and cancel the PLn and EMG noise in ECG signals according to IMFs’ noise magnitude spectrum where the EMD, the EEMD and the grey spectral noise estimation (GSNE) are employed. In the first stage, the EMD decomposes the input ECG signal into IMFs. Then the GSNE is used to estimate noise in IMFs and calculate the related noise energies through its noise magnitude spectrum. By a user-defined threshold, noisy IMFs are identified and put into the second stage. In the second stage, the noisy IMFs are reconstructed and decomposed by the EEMD. Then the IMFs are rechecked in a similar manner to the first stage. If an IMF is considered as noisy, it is discarded. The procedure is repeated for each IMF. At last, the ECG signal is reconstructed with all retained IMF components. To evaluate the performance, a noise energy ratio in dB ($NER_{dB}$) is employed in this paper.

This paper is organized as follows. Section 2 describes the ECG signals with additive noise and the ECG signal decomposition algorithms, the EMD and the EEMD. Section 3 introduces the GSNE based on the first-order grey model of one variable, GM(1,1) model [22,23], and then describes how the GSNE is applied to ECG noise cancellation. Next, the proposed GSNC scheme is introduced in Section 4. In Section 5, the proposed GSNC scheme is justified by forty-three datasets from the MIT-BIH database [24]. Discussions about the EMD, the EEMD and the proposed GSNC are given in Section 6. Finally, the conclusion is given in Section 7.

## 2. The ECG Signal, EMD and EEMD

In this section, the ECG signals with additive noise, the EMD and the EEMD for the ECG signals used in the proposed GSNC scheme are described in the following.

### 2.1. ECG Signal

In this paper, the real ECG signals are from the cardiac arrhythmia database in the MIT-BIH database [24] from which datasets are selected. Each dataset has a continuous period of 30 minutes with the sampling frequency of 360 Hz. The ECG signals in the database were processed by the Butterworth filter with bandwidth from 0.3 Hz to 40 Hz. The processed ECG signal is considered as clean ECG signal, s(k). The PLn and EMG noise are added into s(k) where the PLn is generated from the sinusoidal wave with frequencies from 59.5 Hz to 60.5 Hz and the sampling frequency of 360 Hz. As for the EMG noise, its bandwidth is from 100 Hz to 500 Hz with the sampling frequency 1000 Hz. The additive noise, the PLn or EMG noise, is denoted as n(k). And the noisy ECG signal is denoted as x(k) which is expressed as

(1) x(k)=s(k)+n(k).

In this paper, the signal-to-noise ratio (SNR) from −5 dB to 20 dB is under consideration whose definition is given as

(2) $$SNR=\frac{\sum_{k=1}^{N_{s}}s^{2}\left(k\right)}{\sum_{k=1}^{N_{s}}n^{2}\left(k\right)},$$

where $N_{s}$ is the length of s(k).

### 2.2. The EMD Algorithm

This subsection briefly reviews the EMD which will be applied to decompose an ECG signal in the proposed GSNC scheme. For details, one may consult [15]. The EMD algorithm consists of the following steps.

Step 1. Find the local maxima and minima in x(k).
Step 2. Obtain the upper envelope by the local maxima and the lower envelope by local minima, respectively.
Step 3. Calculate the average of the upper and lower envelops, m(k).
Step 4. Find the difference signal d(k)=x(k)−m(k).
Step 5. Check if d(k) is a zero-average process. If yes, then stop and treat d(k) as the first-order IMF (IMF 1), denoted as $c_{1}\left(k\right)$; otherwise, replace x(k) with d(k) and go back to Step 1.
Step 6. Calculate the residual signal $r\left(k\right)=x\left(k\right)-c_{1}\left(k\right)$.
Step 7. Replace x(k) with r(k) and repeat Step 1 to Step 6 to find the second-order IMF (IMF 2), i.e., $c_{2}\left(k\right)$.
Step 8. Repeat Step 1 to Step 7 till $c_{M}\left(k\right)$ is obtained where M is the total number of IMFs.

After the EMD, the original signal can be expressed as

(3) $$x\left(k\right)=\overset{M}{\underset{i=1}{\sum }}c_{i}\left(k\right)+r\left(k\right),$$

where r(k) is generally considered as $c_{M+1}\left(k\right)$.

### 2.3. The EEMD Algorithm

Here, the EEMD algorithm is briefly reviewed. For details, one may consult [18]. The EEMD algorithm is given in the following.

Step 1. Add a white noise sequence w(k) into the target signal x(k), i.e., $x_{1}\left(k\right)=x\left(k\right)+w\left(k\right)$. In this study, noise with SNR=5 dB is used which will be verified in Section 5.1.
Step 2. Apply the EMD algorithm to decompose $x_{1}\left(k\right)$, as described in Section 2.2.
Step 3. Repeat Steps 1 and 2 until the predefined number of trials, $N_{T}$, is reached. Each trial uses the same noise power level. Then a set of IMF components $c_{ij}\left(k\right)$ is obtained where i is the iteration number and j is the order of IMFs.
Step 4. Calculate the ensemble average of $c_{ij}\left(k\right)$ as follows
  (4) $${\mathrm{EEMD}}_{c_{j}\left(k\right)}=\;\frac{1}{N_{T}}\overset{N_{T}}{\underset{i=1}{\sum }}c_{ij}\left(k\right),$$
  where $N_{T}$ is the total number of trials.

## 3. Application of GSNE to ECG Noise Cancellation

In this section, the noise estimation based on a grey model is introduced. Section 3.1 briefly reviews the first-order grey model with one variable, denoted as GM(1,1). Next, the grey spectral noise estimation (GSNE) based on GM(1,1) model is proposed in Section 3.2. Then the way to determine noisy IMFs by the GSNE is given in Section 3.3.

### 3.1. GM(1,1) Model

In this subsection, GM(1,1) model is briefly reviewed. For details, one may refer [22,23]. The GM(1,1) modeling is described in the following. Given a non-negative sequence {x(k), 1≤k≤K}, then x(k) is put into the first-order accumulated generating operation (1-AGO) to convert into a new data sequence $x^{\left(1\right)}\left(k\right)$ as

(5) $$x^{\left(1\right)}\left(k\right)=\overset{k}{\underset{i=1}{\sum }}x\left(i\right).$$

By x(k) and $x^{\left(1\right)}\left(k\right)$, a grey difference equation is formed as

(6) $$x\left(k\right)+az^{\left(1\right)}\left(k\right)=b,$$

for 2≤k≤K, where parameters a and b are called the developing coefficient and the grey input, respectively; and $z^{\left(1\right)}\left(k\right)$ is the background value and defined as

(7) $$z^{\left(1\right)}\left(k\right)=0.5\left[x^{\left(1\right)}\left(k\right)+x^{\left(1\right)}\left(k-1\right)\right].$$

Let

(8) $$y=\left[\begin{array}{c} x\left(2\right)\\ x\left(3\right)\\ \vdots \\ x\left(K\right) \end{array}\right]\;$$

and

(9) $$B=\left[\begin{array}{ccc} -z^{\left(1\right)}\left(2\right) & & 1\\ -z^{\left(1\right)}\left(3\right) & & 1\\ \vdots \; & \vdots & \\ -z^{\left(1\right)}\left(K\right) & & 1 \end{array}\right]\;$$

Then (6) can be written as

(10) $$y=B\left[\begin{array}{c} a\\ b \end{array}\right]$$

where parameters a and b are found by

(11) $$\left[\begin{array}{c} a\\ b \end{array}\right]={\left(B^{T}B\right)}^{-1}B^{T}y.$$

It can be shown that the solution of $x^{\left(1\right)}(k$) is given as

(12) $$x^{\left(1\right)}\left(k\right)=\left[x\left(1\right)-\frac{b}{a}\right]e^{-a\left(k-1\right)}+\frac{b}{a}\;.$$

By the first-order inverse accumulated generating operation (1-IAGO), the estimate of x(k), $\hat{x}\left(k\right)$, is obtained as

(13) $$\hat{x}\left(k\right)=x^{\left(1\right)}\left(k\right)-x^{\left(1\right)}\left(k-1\right).$$

In the GM (1, 1) modeling, the minimum number of samples is 4, i.e., *K* = 4.

### 3.2. Grey Spectral Noise Estimation

**Figure 1:**
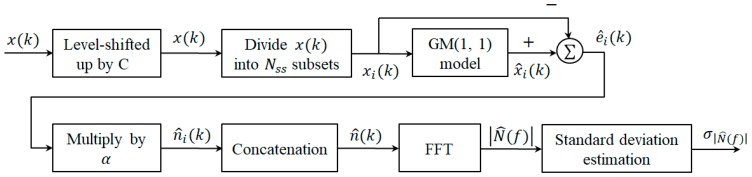
The block diagram of the the grey spectral noise estimation (GSNE).

In this subsection, the grey spectral noise estimation (GSNE) based on GM(1,1) is introduced where an additive signal model is assumed, that is, x(k)=s(k)+n(k) for 1≤k≤L, where s(k), n(k), and L stand for the signal component, noise component and the length of x(k), respectively. The block diagram of GSNE is shown in Figure 1 whose implementation steps are given as follows.

**Figure 2:**
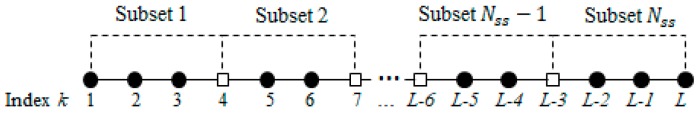
One sample overlapped subsets in the GNSE (K=4).

Step 1. Level up x(k) by a constant C, i.e., x(k)←x(k)+C such that the condition x(k)>0 is met.
Step 2. Divide x(k) into $N_{ss\;}$ subsets as $\left\{x_{i}\left(k\right),\;1\leq i\leq N_{ss\;}\right\}$, for 1+(K−1)(i−1)≤k≤K+(K−1)(i−1) , where *K* is the number of data used in GM(1,1) model. Figure 2 indicates how x(k) is divided into $N_{ss\;}$ subsets with *K* = 4 where the square refers to the overlapped sample.
Step 3. For each subset i, obtain the estimate of $x_{i}\left(k\right)$, ${\hat{x}}_{i}\left(k\right)$, by the GM(1,1) model described in Section 3.1. Then, ${\hat{x}}_{i}\left(k\right)$ is considered as the estimate of $s_{i}\left(k\right)$, ${\hat{s}}_{i}\left(k\right)$, that is, ${\hat{s}}_{i}\left(k\right)={\hat{x}}_{i}\left(k\right)$. Finally, calculate the estimation error of GM(1,1) model as $\;e_{i}\left(k\right)=\;x_{i}\left(k\right)-{\hat{x}}_{i}\left(k\right)$.
Step 4. Since the additive noise $n_{i}\left(k\right)$ is not equal but related to the estimation error $e_{i}\left(k\right)$. Consequently, ${\hat{n}}_{i}\left(k\right)$ is estimated as $\alpha e_{i}\left(k\right)$ where α>0 is the user-defined scaling factor and determined by experiences.
Step 5. Apply Fast Fourier Transform (FFT) on ${\hat{n}}_{i}\left(k\right)$, i.e.,$\;\hat{N}\left(f\right)=\mathrm{FFT}\left\{\hat{n}\left(k\right)\right\}$ and find the magnitude of $\hat{N}\left(f\right)$, $\left|\hat{N}\left(f\right)\right|$ where $\hat{n}\left(1\right)=\hat{n}\left(2\right)$ is used.
Step 6. Calculate the standard deviation of $\left|\hat{N}\left(f\right)\right|$, $\;\sigma_{\left|\hat{N}\left(f\right)\right|}$, as an indicator of noise energy.

Note that the energy preserving property in Parseval’s theorem [25] which proves the sum of the square of ${\hat{n}}_{i}\left(k\right)$, i.e., energy of ${\hat{n}}_{i}\left(k\right)$, is related to the sum of the square of $\left|\hat{N}\left(f\right)\right|$ as

(14) $$\;\overset{L}{\underset{i=1}{\sum }}{\left[\;{\hat{n}}_{i}\left(k\right)\right]}^{2}=\frac{1}{L}\;\overset{L}{\underset{i=1}{\sum }}{\left[\left|\hat{N}\left(f\right)\right|\right]}^{2}.$$

Since the standard deviation $\sigma_{\left|\hat{N}\left(f\right)\right|}$ is a statistics of $\left|\hat{N}\left(f\right)\right|$ related to noise energy, it thus can be used as an indicator of noise energy in the IMF under consideration.

### 3.3. Noisy IMF Determination by the GSNE

**Figure 3:**
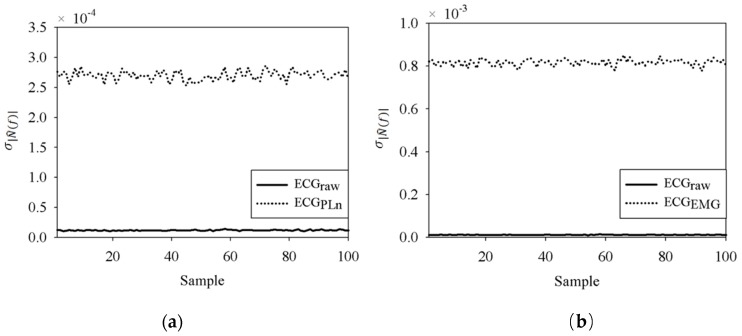
Differences of $\sigma_{\left|\hat{N}\left(f\right)\right|}$ (**a**) ${\mathrm{ECG}}_{\mathrm{raw}}$ vs ${\mathrm{ECG}}_{\mathrm{PLn}}$ (**b**) ${\mathrm{ECG}}_{\mathrm{raw}}$ vs ${\mathrm{ECG}}_{\mathrm{EMG}}$.

In this section, the determination of noisy IMFs and the ECG noise cancellation based on the GSNE are described. In the experiment, we randomly select 100 ECG signal segments with the continuous duration of 10 seconds from the mitdb/100 dataset. Then $\sigma_{\left|\hat{N}\left(f\right)\right|}$ is calculated for each of the ECG signals with and without noises. With SNR=5 dB, Figure 3 shows the differences of $\sigma_{\left|\hat{N}\left(f\right)\right|}$ in IMF 1, i.e., $c_{1}\left(k\right)$, where ${\mathrm{ECG}}_{\mathrm{raw}}$ denotes the original ECG signal,${\;\mathrm{ECG}}_{\mathrm{PLn}}$ the ECG signal with the PLn and ${\mathrm{ECG}}_{\mathrm{EMG}}$ the ECG signal with EMG noise, respectively. As shown in Figure 3, $\sigma_{\left|\hat{N}\left(f\right)\right|}$ with and without noise has apparently different levels. Besides, the fluctuation is very small both for cases with and without noise. Thus, it gives us a hope to discriminate noisy and non-noisy $c_{i}\left(k\right)$ by a predefined threshold τ. According to our experiments, $\tau =10^{-4}$ works well for most cases. If $\sigma_{\left|\hat{N}\left(f\right)\right|}>\tau$, the related $c_{i}\left(k\right)$ is considered as noisy and is rechecked further in the proposed GSNC scheme. With the idea, $c_{i}\left(k\right)$ is modified as ${\bar{c}}_{i}\left(k\right)$ which is given as

(15) $${\bar{c}}_{i}\left(k\right)\;=\{\begin{array}{cc} c_{i}\left(k\right)\; & ,\;\sigma_{\left|\hat{N}\left(f\right)\right|,\;i}<\tau \\ \;0\; & ,\sigma_{\left|\hat{N}\left(f\right)\right|,\;i}>\tau \end{array},$$

for i=1, 2, ⋯,M.

By ${\bar{c}}_{i}\left(k\right)$, the ECG signal after noise cancellation, denoted as $\tilde{y}\left(k\right)$, is reconstructed, as follows,

(16) $$\tilde{y}\left(k\right)=\overset{M}{\underset{i=1}{\sum }}{\bar{c}}_{i}\left(k\right)+r\left(k\right)\;.$$

In order to justify the fixed threshold $\tau =10^{-4}$ works well for most cases, further experiments are conducted with six MIT-BIH cardiac arrhythmia datasets which are mitdb/100, mitdb/105, mitdb/108, mitdb/203, mitdb/223 and mitdb/228. In the experiments, ECG signal segments with the continuous duration of 10 seconds are randomly selected in the six datasets where SNR=5 dB both for PLn and EMG noise. The results are shown in Table 1 for the PLn and Table 2 for the EMG noise, respectively. As shown in Table 1, the $\sigma_{\left|\hat{N}\left(f\right)\right|}$ of x(k) decreases as the order of IMF increases and most cases are determined correctly except for mistaken cases. The noisy IMFs considered as clean are shadowed according to the threshold $\tau =10^{-4}$. The mistaken cases found in IMF 1 are in datasets mitdb/100 and mitdb/223. For those cases, clean IMFs are considered as noisy ones. These mistakes will be rechecked in the second stage of the proposed GSNE scheme described later in Section 4. The mistaken cases found in IMF 4 and IMF 5 consider noisy IMFs as clean ones. The cases are not a major concern since they have little impact on ECG noise cancellation. Thus, they are neglected in this paper.

As for the EMG noise, Table 2 shows most of the clean and noisy cases are determined correctly except some cases. Two clean cases are mistaken as noisy happened in IMF 1 in mitdb/100 and in mitdb/223. For those noisy IMFs, the proposed GSNC scheme introduced later in Section 4 will recheck them further. Similar to the PLn cases, most of the noisy cases are considered as clean found in IMF 4 and IMF 5. Since those cases have little effect on the performance for ECG noise cancellation, thus we do not deal with them further. By the results, shown in Table 1 and Table 2, the fixed threshold $\tau =10^{-4}$ is justified working well for most cases.

## 4. The Proposed GSNC Scheme

**Figure 4:**
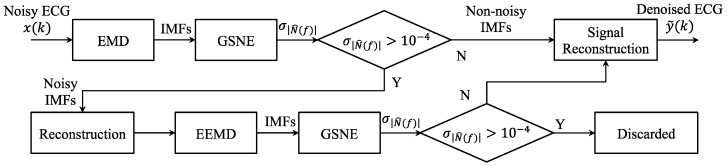
The flowchart of the proposed GSNC scheme.

Figure 4 shows the flowchart of the proposed two-stage ECG noise cancellation scheme based on the GSNE. The proposed scheme is called grey spectral noise cancellation (GSNC). The working flow of the proposed GSNC scheme is described as follows. In the first stage, the input ECG signal is decomposed into a set of IMFs by the EMD. Then the noise spectral energy of IMFs are estimated by the GNSE and the magnitude standard deviation of each noise spectral energy, $\sigma_{\left|\hat{N}\left(f\right)\right|}$, is calculated. Next, $\sigma_{\left|\hat{N}\left(f\right)\right|}$ is compared with a user-defined threshold τ. If $\sigma_{\left|\hat{N}\left(f\right)\right|}>\tau$, the IMF is considered as noisy. Due to the IMFs may mix each other, the second stage is performed to avoid mistakes. In the second stage, the noisy IMFs considered in the first stage are reconstructed and decomposed by the EEMD. The decomposed IMFs are then checked by the GNSE as in the first stage. If $\sigma_{\left|\hat{N}\left(f\right)\right|}>\tau$, the corresponding IMF is discriminated as noisy and discarded. Finally, the ECG is reconstructed with all retained IMFs. This finishes the procedure.

To evaluate the performance of the proposed GSNC scheme, a performance index to evaluate the improvement based on the noise energy ratio in dB ($NER_{dB}$) is employed which is defined as

(17) $$NER_{dB}=10\log\left[\frac{\sum_{k=1}^{N_{s}}{\left(x\left(k\right)-s\left(k\right)\right)}^{2}}{\sum_{k=1}^{N_{s}}{\left(\tilde{y}\left(k\right)-s\left(k\right)\right)}^{2}}\right],\;$$

where s(k) is the true ECG signal; x(k) and $\tilde{y}\left(k\right)$ are the input noisy ECG signal and the reconstructed ECG signal after noise cancellation; $N_{s}$ is the total number of samples in x(k).

## 5. Results 

In this section, the proposed GSNC scheme is justified by forty-three datasets from the MIT-BIH cardiac arrhythmia database. In Section 5.1, the energy of white noise added in the EEMD is investigated. In Section 5.2, two types of noise, the PLn and EMG noise, are considered in the experiments.

### 5.1. Effect of White Noise in the EEMD

**Figure 5:**
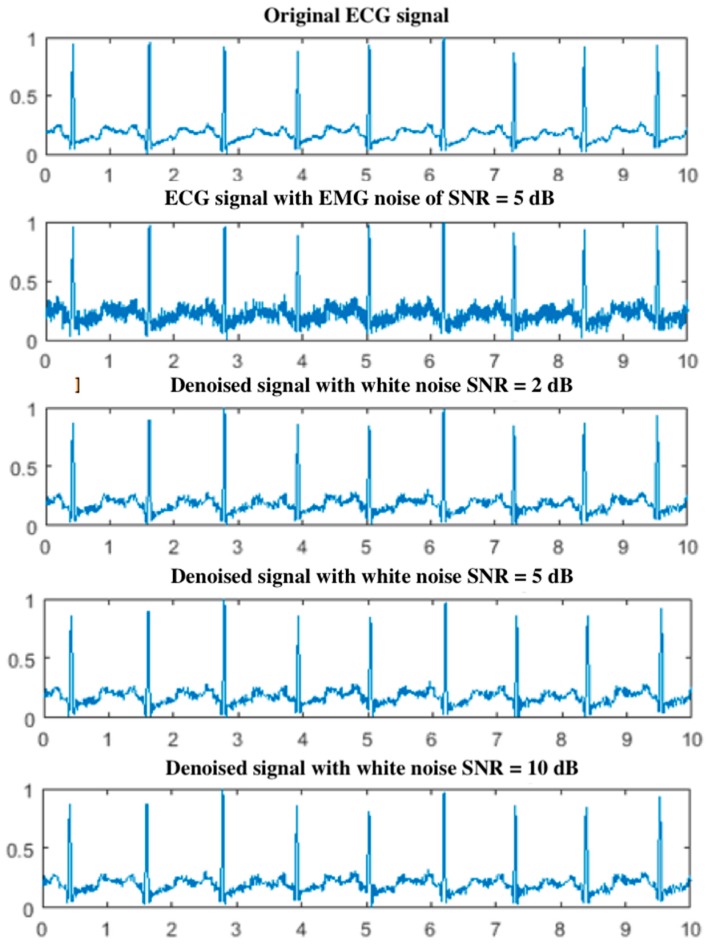
The original ECG signal and ECG signals after the proposed GSNC scheme with different SNR of white noise in the ensemble empirical mode decomposition (EEMD) (EMG noise, SNR=5 dB).

In this subsection, the effect of white noise energy on the EEMD employed in the proposed GSNC is investigated. It is observed that the energy of white noise added in the EEMD affects the performance of noise cancellation. Consequently, several levels of white noise energy are added into the EEMD to find an appropriate one. In the experiments, three SNR used in the EEMD are 2 dB, 5 dB and 10 dB where an ECG signal with EMG noise of SNR=5 dB is under consideration. Besides, the number of trials $N_{T}=100$ is employed in the EEMD. Figure 5 shows parts of the original ECG signal and the ECG signals after noise cancellation by the proposed GSNC scheme. The corresponding $NER_{dB}$ are 7.84 dB, 7.92 dB and 5.26 dB for the cases with SNR=2 dB, 5 dB and 10 dB in the EEMD, respectively. By the $NER_{dB}$, it suggests that the white noise with SNR=5 dB results in the best performance of ECG noise cancellation. Consequently, it is employed in the EEMD which is applied in the second stage of the proposed GSNC for better performance.

### 5.2. Results for the PLn and EMG Noise

The PLn and EMG noise are generated artificially and added into the original ECG signals whose SNR=5 dB and all forty-three MIT-BIH datasets are involved. As for the GSNE, the parameters K=4 and α=1 are set. In the experiment, the proposed GSNC scheme is also compared with the traditional noise cancellation schemes based on the EMD and the EEMD. As with the first experiment, the PLn with SNR=5 dB is considered. Table 3 shows the $NER_{dB}$ for the proposed GSNC and compared schemes. In Table 3, the EMD scheme has the $NER_{dB}$ ranged from−12.83 dB to 7.96 dB with the largest standard deviation of 5.21 dB among the compared schemes. For the EEMD scheme, its $NER_{dB}$ ranges from −5.67 dB to 7.90 dB in the given datasets with a smallest standard deviation of 3.21 dB. As for the proposed GSNC scheme, its $NER_{dB}$ ranges from −5.33 dB to 9.97 dB. On the average of $NER_{dB}$, the proposed GSNC scheme has the best result (3.34 ± 4.03 dB) which is followed by the EEMD (2.20 ± 3.21 dB) and EMD (0.10 ± 5.21 dB) schemes.

In the second experiment, the EMG noise of SNR=5 dB is under study. Table 4 gives the $NER_{dB}$ for the proposed GSNC, EMD and EEMD schemes. In this experiment, a similar result to the first experiment is found. In the EMD scheme, the $NER_{dB}$ ranges from −8.03 dB to 12.19 dB with the largest standard deviation of 4.52 dB. The EEMD scheme has the range of $NER_{dB}$ from −6.66 dB to 12.47 dB with the smallest standard deviation of 4.27 dB. And the $NER_{dB}$ for the proposed GSNC scheme is within −5.14 dB to 13.21 dB. On the average of $NER_{dB}$, the proposed GSNC scheme has the highest value (6.14 ± 4.29 dB) followed by the EEMD scheme (3.85 ± 4.27 dB) and the EMD scheme (3.52 ± 4.52 dB). 

**Figure 6:**
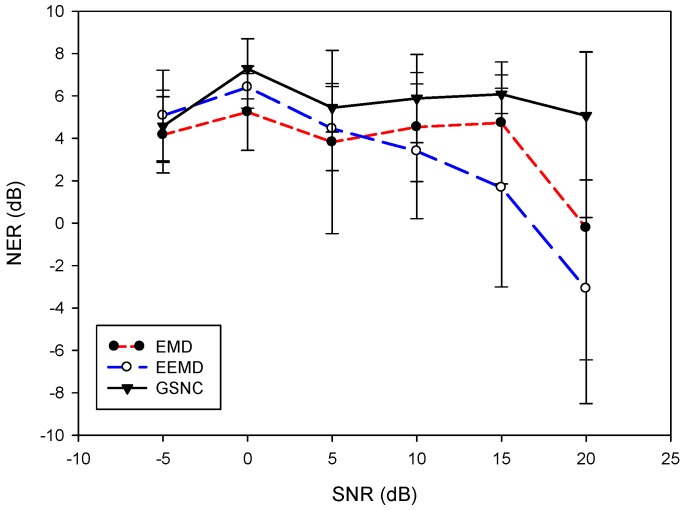
Comparison of overall average $NER_{dB}$ with various *SNR* for the proposed GSNC, EMD and EEMD schemes (PLn).

To investigate the proposed GSNC scheme further, the ECG signal with various noise energies in the PLn and EMG noise are under consideration. The SNR for the PLn and EMG noise used in the experiments are −5 dB, 0 dB, 5 dB, 10 dB, 15 dB and 20 dB. Six datasets from MIT-BIH database are selected, including mitdb/100, mitdb/105, mitdb/108, mitdb/203, mitdb/223 and mitdb/228. For the case of PLn, Figure 6 shows the overall average of $NER_{dB}$ for the proposed GSNC scheme and the compared EMD and EEMD schemes. On the average of $NER_{dB}$ for all cases with the corresponding standard deviation, the EMD scheme obtains 4.16 ± 1.79 dB, 5.24 ± 1.81 dB, 3.82 ± 4.32 dB, 4.53 ± 2.57 dB, 4.72 ± 2.88 dB and −0.22 ± 8.29 dB for SNR= −5 dB, 0 dB, 5 dB, 10 dB, 15 dB and 20 dB, respectively. The results for the EEMD scheme are 5.07 ± 2.13 dB, 6.41 ± 0.99 dB, 4.46 ± 1.98 dB, 3.39 ± 3.17 dB, 1.67 ± 4.68 dB and −3.09 ± 3.36 dB for SNR= −5 dB, 0 dB, 5 dB, 10 dB, 15 dB and 20 dB, respectively. And the proposed GSNC scheme has 4.5 ±1.69 dB, 7.28 ± 1.41 dB, 5.44 ± 1.14 dB, 5.88 ± 2.07 dB, 6.08± 0.91 dB and 5.07 ± 3.02 dB for SNR= −5 dB, 0 dB, 5 dB, 10 dB, 15 dB and 20 dB, respectively. As shown in Figure 6, the proposed GSNC scheme always has better $NER_{dB}$ than the compared schemes except the case SNR=−5 dB.

**Figure 7:**
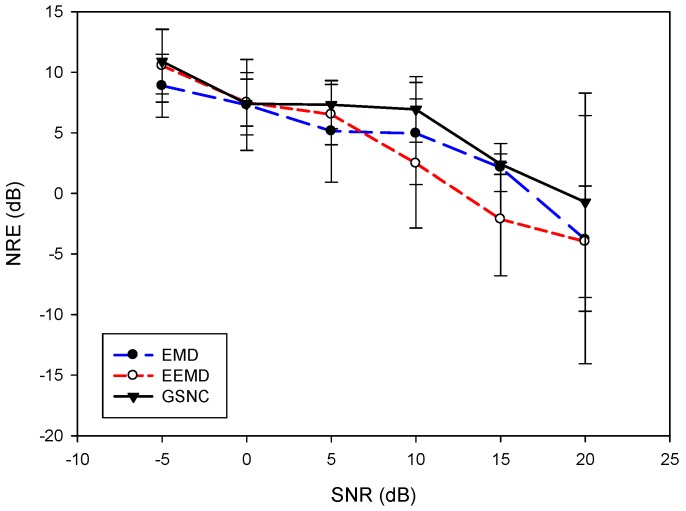
Comparison of overall average $NER_{dB}$ with various *SNR* for the proposed GSNC, EMD and EEMD schemes (EMG noise).

In the case of EMG noise, Figure 7 depicts the averages of $NER_{dB}$ for different SNR. The $NER_{dB}$ and standard deviations for the EMD scheme are 8.88 ± 2.59 dB, 7.29 ± 3.75 dB, 5.13 ± 4.20 dB, 4.94 ± 4.21 dB, 2.13 ± 1.97 dB and −3.81 ± 10.24 dB for SNR −5 dB, 0 dB, 5 dB, 10 dB, 15 dB and 20 dB, respectively. For the EEMD scheme, the average $NER_{dB}$ with corresponding standard deviations are 10.52 ± 7.50 dB, 7.50 ± 1.93 dB, 6.50 ± 2.48 dB, 2.46 ± 5.32 dB, −2.13 ± 4.66 dB and −3.98 ± 4.56 dB for SNR= −5 dB, 0 dB, 5 dB, 10 dB, 15 dB and 20 dB, respectively. The results for the proposed GSNC scheme are 10.89 ± 2.68 dB, 7.40 ± 2.56 dB, 7.32 ± 1.97 dB, 6.93 ± 2.70 dB, 2.41 ± 0.84 dB and −0.72 ± 9.0 dB for SNR= −5 dB, 0 dB, 5 dB, 10 dB, 15 dB and 20 dB, respectively. For all cases, the proposed GSNC scheme is superior to the compared schemes by $NER_{dB}$ except slightly less than the EEMD scheme in the case of SNR=0 dB. By Figure 6 and Figure 7, it suggests that the proposed GSNC scheme generally shows better performance when compared with the EMD and EEMD schemes in terms of average $NER_{dB}$, both for the PLn and EMG noise.

## 6. Discussion

The EMD acts like a filter-bank and has no strict bandwidth restriction with the IMFs. The frequency range of each IMF is adaptive, depending on the original signal content. Generally, the bandwidths of the PLn and EMG noise are assumed in the ranges 59.5–60.5 Hz and 100–500 Hz, respectively. Thus the PLn is considered as a lower frequency noise while the EMG noise is considered as a medium and higher frequency noise. Note that the noise energy is mainly distributed in the low order IMF components after the EMD. Because of different bandwidths, the performance for the EMG noise is better than that for the PLn in the EMD scheme for ECG noise cancellation. As shown in Table 3 and Table 4, the average $NER_{dB}$ for the PLn is 0.10 dB and 3.52 dB for the EMG noise.

Compared with the EMD, the EEMD has more concentrated band-limit IMF components. With the iterative EMD computation, the average of IMF with the same order yielded a sharper band transition than a single EMD-derived IMF, that is, the transition band overlap between adjacent IMFs is narrower than the EMD result. In other words, with the same filter specification the EEMD acts like a higher-order filter while the EMD works like a lower-order filter. Consequently, the performance of the EEMD scheme is better than the EMD scheme, as shown in Table 3 and Table 4 where the average $NER_{dB}$ for PLn and the EMG noise by the EEMD scheme are 2.20 dB and 3.85 dB, respectively. They are higher than the corresponding $NER_{dB}$ by the EMD scheme. However, the EEMD pays the price of computational complexity, that is, it takes more time to cancel the ECG noise. This hinders the realization in an ECG patch if only the EEMD is applied in the ECG noise cancellation. On contrarily, the proposed GSNC scheme decomposes the input ECG signal by the EMD in the first stage. When $\sigma_{\left|\hat{N}\left(f\right)\right|,\;i}<\tau$, the proposed GSNC scheme stops further decomposition. This makes the proposed GSNC scheme possible to be embedded in an ECG patch. Moreover, the proposed GSNC scheme employs two-stage discrimination for noisy IMFs while the conventional EMD and EEMD schemes use one-stage discrimination. Thus, the proposed GSNC scheme is expected to have better performance since suspicious IMFs in the first stage can be rechecked in the second stage to avoid mistakes while the one-stage EMD and EEMD scheme fails to.

In [17], Liu et al. showed that the energy of a lower frequency in the EMG noise is found in the first-order IMF component. In other words, the EMD or the EEMD like a filter-bank is able to decompose different intrinsic components in an ECG signal. This is also true for the PLn case. The results. shown in Table 1 and Table 2 have justified the idea where the IMFs for clean and noisy ECG signals can be discriminated in most cases. Another evidence is in Table 3 and Table 4. The results indicate that the average $NER_{dB}$ are positive. That is, intrinsic noise and additive noise can be dealt and cancelled in the EMD, the EEMD and the proposed GSNC schemes. Among the three schemes, the proposed GSNC scheme has the best performance with the average $NER_{dB}$ 3.34 dB for the PLn and 6.14 dB for the EMG noise, as shown in Table 3 and Table 4, respectively. It implies that the proposed GSNC scheme is able to estimate the noise appropriately and the two-stage discrimination can relieve the over-cancellation problem which generally happens in the one-stage discrimination.

**Figure 8:**
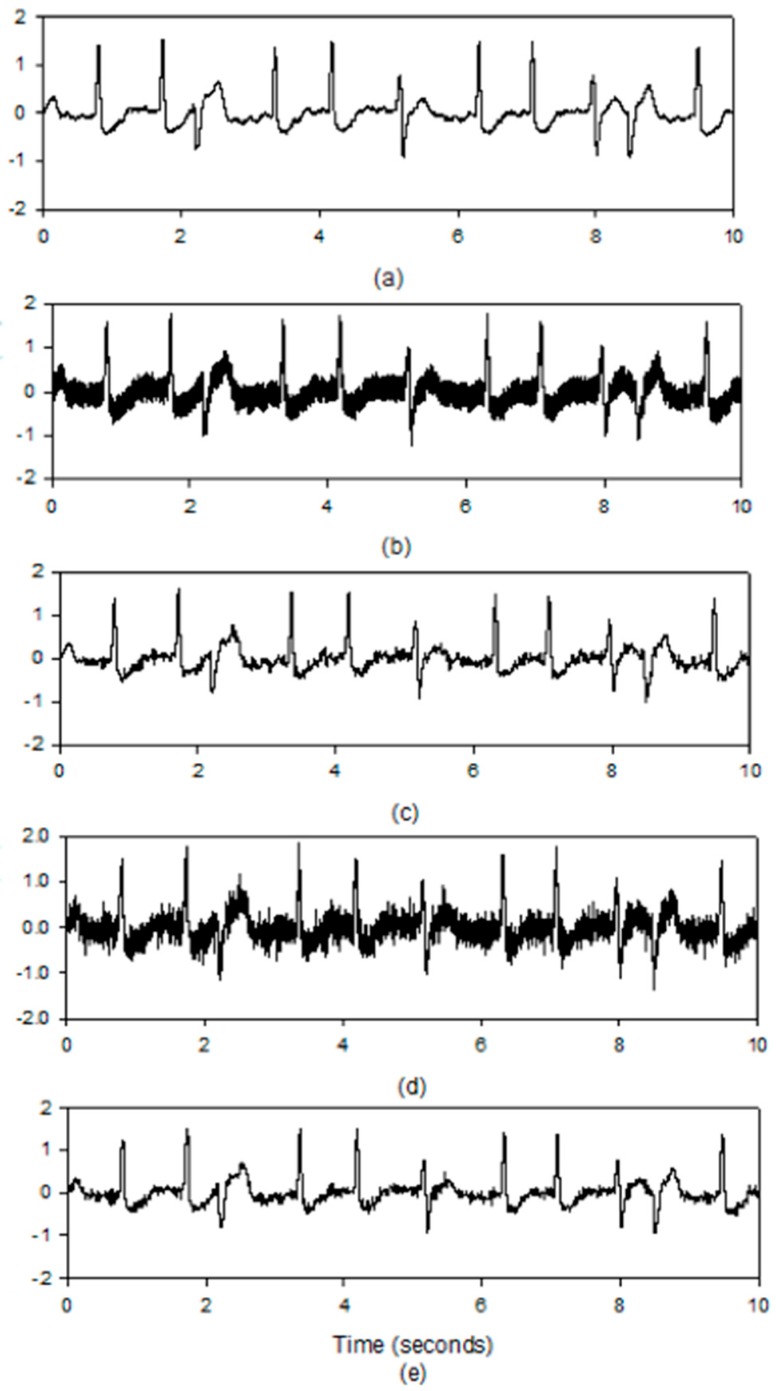
The arrhythmic ECG signals, (**a**) the original ECG signal with two PVC beats, (**b**) the ECG with PLn, (**c**) the denoised ECG with PLn, (**d**) the ECG with EMG noise (**e**) the denoised ECG with EMG noise.

In order to show the proposed GSNC scheme does not affect the morphology of arrhythmic beat, the arrhythmic ECG dataset mitdb/210 is given as an example where two premature ventricular contraction (PVC) beats are within the duration. The denoised results for the PLn and EMG noise (SNR=5 dB) by the proposed GSNC scheme are given in Figure 8. As shown in Figure 8b,c, the proposed GSNC scheme is able to retain subtle signs in the denoised ECG signal, when compared with Figure 8a.

## 7. Conclusions

This paper has presented a grey spectral noise cancellation (GSNC) scheme for ECG signals. In the proposed GSNC scheme, two-stage discrimination for noisy IMFs was employed which included the EMD, the EEMD and grey spectral noise estimation (GSNE). In general condition, the lower order IMFs would be easily coupled with the noise. The GSNE was applied to estimate the noise energy through the standard deviation of noise magnitude spectrum $\sigma_{\left|\hat{N}\left(f\right)\right|}$. By $\sigma_{\left|\hat{N}\left(f\right)\right|}$, noisy IMFs were determined and discarded in the process of reconstruction. The proposed GSNC scheme has been verified by forty-three datasets from the MIT-BIH database where different SNR levels for the PLn and EMG noise were considered. The results indicated that the proposed GSNC scheme was generally superior to the compared EMD and EEMD schemes in terms of average $NER_{dB}$ in the given datasets. The proposed GNSC scheme provides a new approach, based on the noise magnitude spectrum, to estimate the noise energy in the IMFs. The proposed GNSC scheme could be implemented in an embedded system, like the ECG patch, to deal with noises in the ECG signals.

## Figures and Tables

**Table 1:** The $\sigma_{\left|\hat{N}\left(f\right)\right|}$ for clean signal s(k) and noisy signal x(k) with PLn (SNR=5 dB).

		mitdb/100	mitdb/105	mitdb/108	mitdb/203	mitdb/223	mitdb/228
IMF 1	s(k)	1.16 × 10^−4^	7.90 × 10^−5^	6.97 × 10^−5^	8.37 × 10^−5^	1.27 × 10^−4^	7.84 × 10^−5^
	x(k)	2.56 × 10^−3^	4.54 × 10^−3^	2.78 × 10^−3^	4.40 × 10^−3^	6.39 × 10^−3^	2.60 × 10^−3^
IMF 2	s(k)	5.18 × 10^−5^	3.91 × 10^−5^	2.93 × 10^−5^	4.23 × 10^−5^	5.065 × 10^−5^	3.24 × 10^−5^
	x(k)	6.58 × 10^−4^	1.22 × 10^−3^	6.52 × 10^−4^	1.13 × 10^−3^	1.61 × 10^−3^	6.81 × 10^−4^
IMF 3	s(k)	2.66 × 10^−5^	3.05 × 10^−5^	1.70 × 10^−5^	2.37 × 10^−5^	2.98 × 10^−5^	1.57 × 10^−5^
	x(k)	2.08 × 10^−4^	3.51 × 10^−4^	2.06 × 10^−4^	3.31 × 10^−4^	4.85 × 10^−4^	2.02 × 10^−4^
IMF 4	s(k)	1.43 × 10^−5^	1.36 × 10^−5^	9.01 × 10^−6^	1.25 × 10^−5^	2.51 × 10^−5^	7.02 × 10^−6^
	x(k)	8.93 × 10^−5^	1.01 × 10^−4^	6.09 × 10^−5^	1.05 × 10^−4^	1.65 × 10^−4^	5.63 × 10^−5^
IMF 5	s(k)	6.58 × 10^−6^	6.85 × 10^−6^	5.22 × 10^−6^	7.67 × 10^−6^	1.12 × 10^−5^	3.80 × 10^−6^
	x(k)	3.28 × 10^−5^	4.26 × 10^−5^	2.40 × 10^−5^	3.59 × 10^−5^	6.25 × 10^−5^	2.61 × 10^−5^

**Table 2:** The $\sigma_{\left|\hat{N}\left(f\right)\right|}$ for clean signal s(k) and noisy signal x(k) with EMG noise (SNR=5 dB).

		mitdb/100	mitdb/105	mitdb/108	mitdb/203	mitdb/223	mitdb/228
IMF 1	s(k)	1.16 × 10^−4^	7.90 × 10^−5^	6.97 × 10^−5^	8.37 × 10^−5^	1.27 × 10^−4^	7.84 × 10^−5^
	x(k)	1.36 × 10^−3^	2.51 × 10^−3^	1.43 × 10^−3^	2.37 × 10^−3^	3.42 × 10^−3^	1.37 × 10^−3^
IMF 2	s(k)	5.18 × 10^−5^	3.91 × 10^−5^	2.93 × 10^−5^	4.23 × 10^−5^	5.06 × 10^−5^	3.24 × 10^−5^
	x(k)	4.00 × 10^−4^	7.75 × 10^−4^	4.75 × 10^−4^	7.04 × 10^−4^	1.03 × 10^−3^	4.82 × 10^−4^
IMF 3	s(k)	2.66 × 10^−5^	3.05 × 10^−5^	1.70 × 10^−5^	2.37 × 10^−5^	2.98 × 10^−5^	1.57 × 10^−5^
	x(k)	1.40 × 10^−4^	2.06 × 10^−4^	1.30 × 10^−4^	2.09 × 10^−4^	3.17 × 10^−4^	1.20 × 10^−4^
IMF 4	s(k)	1.43 × 10^−5^	1.36 × 10^−5^	9.01 × 10^−6^	1.25 × 10^−5^	2.5 × 10^−5^	7.02 × 10^−6^
	x(k)	5.53 × 10^−5^	6.60 × 10^−5^	3.93 × 10^−5^	5.89 × 10^−5^	9.61 × 10^−5^	3.92 × 10^−5^
IMF 5	s(k)	6.58 × 10^−6^	6.85 × 10^−6^	5.22 × 10^−6^	7.67 × 10^−6^	1.12 × 10^−5^	3.80 × 10^−6^
	x(k)	2.14 × 10^−5^	3.13 × 10^−5^	1.28 × 10^−5^	2.14 × 10^−5^	3.65 × 10^−5^	1.52 × 10^−5^

**Table 3:** The $NER_{dB}$ for the proposed GSNC and the compared schemes with the PLn (SNR=5 dB).

Dataset	EMD	EEMD	GSNC	Dataset	EMD	EEMD	GSNC
mitdb/100	−4.59	6.36	6.51	mitdb/205	1.59	−1.14	5.08
mitdb/101	1.01	0.32	2.83	mitdb/207	4.12	6.92	9.48
mitdb/103	−1.72	1.09	1.17	mitdb/208	−4.45	2.79	−2.07
mitdb/105	6.04	3.53	5.02	mitdb/209	−7.96	−0.60	−5.02
mitdb/106	−5.32	0.12	−1.48	mitdb/210	6.58	4.24	7.68
mitdb/108	3.29	6.52	3.50	mitdb/212	−11.33	−3.40	−5.33
mitdb/109	7.96	7.90	9.97	mitdb/213	3.97	−3.15	7.10
mitdb/111	4.19	1.53	5.48	mitdb/214	−2.72	5.24	−0.91
mitdb/112	6.75	5.24	7.52	mitdb/215	−2.11	0.08	1.22
mitdb/113	−2.12	−1.81	−0.72	mitdb/219	−8.31	5.23	2.91
mitdb/114	−0.75	2.78	2.99	mitdb/220	−1.13	−0.52	0.40
mitdb/115	−3.01	−1.50	0.10	mitdb/221	2.23	2.87	3.46
mitdb/116	−3.52	3.77	3.32	mitdb/222	0.97	−0.39	6.22
mitdb/117	3.11	0.31	5.65	mitdb/223	5.48	5.62	6.24
mitdb/118	−1.84	−0.53	−0.19	mitdb/228	7.31	1.67	6.27
mitdb/119	2.03	0.37	3.32	mitdb/230	−1.58	0.32	−0.26
mitdb/121	2.76	2.83	3.88	mitdb/231	−6.55	−5.67	−4.45
mitdb/122	5.46	4.18	9.73	mitdb/232	−2.65	6.47	5.64
mitdb/123	−0.52	−0.42	−2.01	mitdb/233	0.73	6.18	6.68
mitdb/124	5.82	5.61	6.74	mitdb/234	−5.21	0.66	−0.22
mitdb/201	−12.83	5.44	6.72				
mitdb/202	7.83	4.53	8.42	Mean	0.10	2.20	3.34
mitdb/203	5.40	3.07	5.15	Std.	5.21	3.21	4.03

**Table 4:** The $NER_{dB}$ for the proposed GSNC and the compared schemes with the EMG noise (SNR=5 dB).

Dataset	EMD	EEMD	GSNC	Dataset	EMD	EEMD	GSNC
mitdb/100	−3.02	8.66	7.94	mitdb/205	1.71	1.91	10.87
mitdb/101	0.14	2.18	2.65	mitdb/207	11.32	12.47	13.21
mitdb/103	−2.79	1.70	0.77	mitdb/208	−2.11	6.44	2.81
mitdb/105	6.44	7.68	8.21	mitdb/209	0.31	−1.44	1.65
mitdb/106	5.03	2.21	5.42	mitdb/210	7.79	3.66	9.50
mitdb/108	6.61	7.39	3.87	mitdb/212	−1.29	−4.15	−5.14
mitdb/109	10.84	11.17	12.61	mitdb/213	2.55	7.52	5.96
mitdb/111	8.23	3.79	9.44	mitdb/214	7.64	4.59	10.04
mitdb/112	1.50	3.16	1.97	mitdb/215	2.13	0.14	3.66
mitdb/113	−0.92	1.76	1.43	mitdb/219	7.62	4.16	9.44
mitdb/114	4.96	9.50	12.16	mitdb/220	0.42	−1.53	1.11
mitdb/115	1.43	−2.90	−0.77	mitdb/221	4.54	5.95	6.93
mitdb/116	3.27	6.24	9.45	mitdb/222	5.32	3.54	7.06
mitdb/117	−0.53	−0.08	6.12	mitdb/223	6.30	8.59	7.63
mitdb/118	−1.63	−1.98	−0.57	mitdb/228	5.25	3.47	6.55
mitdb/119	7.59	1.12	8.68	mitdb/230	0.82	0.27	3.21
mitdb/121	12.19	9.67	9.94	mitdb/231	−2.91	−6.66	−0.49
mitdb/122	2.73	2.50	5.21	mitdb/232	−8.03	7.15	7.64
mitdb/123	2.86	−1.12	3.38	mitdb/233	8.98	7.05	11.14
mitdb/124	8.53	6.74	11.42	mitdb/234	−0.39	4.08	2.93
mitdb/201	6.67	7.81	9.34				
mitdb/202	4.10	7.98	9.79	Mean	3.52	3.85	6.14
mitdb/203	9.21	3.26	9.72	Std.	4.52	4.27	4.29
